# Book Reviews

**Published:** 1821-08

**Authors:** 


### CRITICAL ANALYSES

OF

### RECENT PUBLICATIONS, IN THE DIFFERENT BRANCHES OF MEDICINE AND SURGERY.

---

"I would have men know, that, though I reprehend the easie passing over of the causes of things "by ascribing them to secret and hidden vertues and properties; (for this hath arrested and laid "asleepe all true enquiry and indications;) yet I doe not understand but that, in the practical "part of knowledge, much will be left to experience and probation, whereunto indication cannot "so fully reach: and this not only in *specie*, but in *individuo*. Yet it was well said, *Vere scire* "*esse per causas scire*."—BACON.

---

*A Treatise on Indigestion, and its Consequences, called Nervous and Bilious Complaints; with Observations on the Organic Diseases in which they sometimes terminate.* By A. P. W. Philip, M.D. F.R.S. ED. &c. 8vo. pp. 363. T. and G. Underwood, London, 1821.

[In continuation from page 148.]

THE author, in conformity with his pathology, treats of the means of cure of what he regards as the first and second stages of indigestion, respectively, in a distinct manner. We have already shown that he considers "indigestion" to be essentially a disease from debility, which is accompanied in the second stage with inflammation. His mode of treating it agrees with these principles. The medicines he recommends for the first stage, are of the stimulative kind: his prophylactic measures are directed with the view of averting the supervention of irritation. His remarks on the diet and regimen proper for those purposes are, for the most part, judicious and interesting, especially those on diet; and, as we always endeavour to support our assertions by cited evidence, we shall adduce some of the most remarkable of them, as we pass them in review in a particular manner. Before we commence, we should however observe, that we regard the remedial measures proposed by the author for the "first stage," in a favourable manner only as they are applicable to indigestion from debility, when inflammation of the stomach is not present. That indigestion sometimes, and not unfrequently, originates with chronic inflammation of the mucous membrane of the stomach, we suppose no one will be adverse to acknowledge: Dr. Philip must be aware of this fact; and we do not conceive why he has neglected to notice it in a precise manner. The whole tenor of his history of the first stage of indigestion, and of the means of treating it, would imply that it arises only from *debility* of the stomach. He does not, it is true, profess to give a complete history of indigestion; but, with this reservation, it would, we think, have been prudent to have stated more precisely that one species of indigestion alone was the subject of his consideration.

Having noticed, in a general way, the importance of due exercise of body and mental quietude, both in a remedial and prophylactic point of view, the author considers the subject of diet. The objects to be kept in view in regulating the diet, he says, are "that it shall tend as little as possible to produce either morbid distention or morbid irritation of the surface of the alimentary canal." Although these matters may appear so simple, and are so well understood by brute animals, a great deal may be said about them, that daily experience proves to be of much importance. This we think ourselves, though we are not much disposed to conform to the fashion of the day, and pretend to be very knowing in what is and what is not good for a stomach, or think that the caprices of this organ, above all others in the body, are to be humoured with so much condescension as is generally showed towards them. We have a friend, who, tired of consulting them in vain, for they varied incessantly, resolved that his stomach should digest what he chose to put into it, or serve him as a stomach no longer, and, after two or three squeamish fits and a week's starvation, managed somewhat like that of Governor Sancho Pança, the organ would do its office very well, and has since served him in a becoming manner. We suspect that the cases of indigestion are not few that might be cured by similar measures; and we recommend them as worthy of a place with the advice of MONTAIGNE for the management of the still more vexatious caprices of another organ.

"All undigested food," the author says, "however small the quantity, is a cause of irritation. Thus, the whole train of symptoms which constitute a fit of indigestion, may arise either from too large a quantity of food, particularly if carelessly masticated, or from food of difficult digestion; most readily, of course, from a combination of these causes. It is therefore of great consequence, in regulating the treatment of this disease, to ascertain what kinds of food are most easily changed by the gastric fluid. This is sometimes influenced by peculiarities of constitution, to which no general rules will apply; but it is not difficult to perceive what kind of diet is generally best suited to a weak stomach.

"Acescent and oily articles of food, with a large proportion of liquid, compose the diet most difficult of digestion. It would appear, that a feeble gastric fluid, as indeed we might *à priori* suppose, does not admit of being greatly diluted, without having its powers much impaired. The diet opposite to this, then, is that which agrees best with dyspeptics. In the first stage of indigestion, a diet composed pretty much of animal food and stale bread is the best."

If we except beef and veal, the author adds, the flesh of old, is generally more easy of digestion than that of young, animals, on account, he says, of the greater quantity of mucilage in the latter; and "all mucilages are of difficult digestion."

"The stronger kinds of animal food, of which beef may be considered the strongest, are most apt to excite fever. On this account, we often allow those recovering from fever, or otherwise disposed to it, to eat the animal mucilages, or those meats which contain a great proportion of them, when even mutton, for example, is forbidden. Thus, animal jellies and young meats have obtained the name of light; but this only relates to the tendency to produce fever, for, as far as digestion is concerned, they are heavier than mutton, and, to many stomachs, than beef. A similar observation applies to the vegetable, compared with the animal, kingdom; the former are less apt to excite fever, and are therefore called lighter, but they are in general more difficult of digestion."

These remarks coincide exactly with the observations of Dr. Lallemande, (noticed in the Number of this Journal for December, 1818,) on subjects having preternatural anus, formed in some part of the small intestines, where there existed peculiarly favourable occasions for acquiring accurate information. There is one important additional fact, noticed by the physician just named, which we should adduce, that of vegetable substances, as pieces of potato, rice, &c. passing almost unaltered from the stomach to the duodenum; whilst meat, taken at the same time, was retained and digested. These facts have a striking relation to the comparative length of the digestive canal in carnivorous and herbivorous animals; the existence of gizzards in granivorous birds, and of several stomachs in herbivorous animals; the great muscularity of the digestive organs in these animals, &c. Duration of the process of digestion has been, by many writers, confounded with difficulty, or regarded as a standard of the direct ratio of the difficulty; but this is erroneous. The duration of digestion, in a healthy stomach, for the most part, is proportionate to the quantity of nutriment contained in a given volume of food. The substances which contain the greatest proportion of nutriment are retained the longest in the stomach. Substances of very difficult digestion, and such as are wholly indigestible, are, for the most part, soon expelled from the stomach, by one orifice or the other, at the same time that substances more proper for digestion are retained. We are aware of some apparent, but not real, objections to this statement. They are furnished by persons of very strong digestive powers, where the relation of difficulty of digestion does not exist. If the food chosen in preference by the languid inhabitants of the torrid zone is urged in objection, it may be replied, that the digestive powers here vary; the properties of the stomach and its fluids have different relations to particular species of aliment. We even find some appearance of this relative change in the structure of parts more distantly related with digestion, as the teeth. The

Tartar, feasting on raw horse-flesh in the icy plains of Siberia, has sharp, scraggy teeth, almost like those of a purely carnivorous animal; whilst the negro, making his diet of fruits and grain, like the apes, his ancient companions of the forest, shaded from the burning skies of the torrid zone by the banana or wide-spreading palm-tree, has large, close-set, and smooth teeth, similar to those of the purely granivorous tribes. It is worthy of remark, that, though there are certain nations where vegetable food alone is generally used, it is not the less evident that animal food is better appropriated for the human body. At Otaheite, the chiefs solely are allowed, by a law of the country, to add a proportion of pork to the fruits and vegetables forming the whole diet of the rest of the people; and they have, in consequence of it, acquired a superiority of stature and vigour, mental as well as bodily, which gives them the character of a different race from the rest of their nation.

Fish, the author observes, is, for the most part, less easy of digestion than the flesh of land animals: "although," he adds, "from the white kinds being less apt to excite fever, (to stimulate the stomach, we should say,) they, like the animal mucilages, have obtained the name of light; a term which so often deceives with respect to what is most easy of digestion, that it is necessary to keep this explanation of it in view." Of all meat, Dr. Philip says, the lean part of venison is, perhaps, the most digestible. Mutton he places next in order of digestibility, though hare and partridge are considered by him to be as easily digestible as mutton. Pork, he remarks, is of comparatively difficult digestion. This observation, and that respecting the digestibility of fish, are in direct opposition to the results of some experiments on dogs, and, amongst them, those of Mr. Astley Cooper; but they seem to be conformable with ordinary experience relative to man. It will be remarked, that the order of aliments as to facility of digestion, is very nearly, or exactly, in a direct ratio to the proportion of azote in them; which, it is well known, is the substance which most contributes to the nutrition of the superior orders of animals. Eggs are considered by Dr. Philip "as of a middle nature between animal and vegetable food." Where not more than one or two is taken, soft-boiled and eaten with stale bread, they are easily digestible. "Few things are of more difficult digestion than new bread. This observation applies to every thing which by mastication forms a tenacious paste, which is not easily pervaded by the gastric fluid." "Even bread sufficiently old," the author adds, "is oppressive, if taken alone and in large quantity; it still forms a mass not very easily pervaded." Labouring people of strong digestive powers have ascertained this fact, and know how to profit by it. The peasants of Holland, before they set out on a long journey in winter, made by skaiting on their canals, eat a large quantity of coarse brown bread, as the food which will keep the digestive function of the stomach the longest employed; and they choose this because, when their stomach becomes empty, they begin to feel pain about the epigastrium, and get fatigued. The English labourer prefers pork to other meats, for the same reason.

Having considered the relative digestibility of different sorts of animal food, the author makes a few remarks on the means of cooking it. " Our food," he says, " is rendered more easy of digestion by simply roasting or boiling, provided it is not too much done. Beyond this, the art of cookery is nothing, but that of pleasing the palate at the expence of the stomach." It is a curious thing that cookery is an art so generally resorted to by man; it is, indeed, almost universal; for, if we except some of the Tartaric and Scythian tribes,—and most of those do not eat their horse-flesh quite raw, but stew it under their saddles,—the Icthyophagists of the eastern coast of Africa, and some of the more savage tribes of the interior of this quarter of the world, who only suffer their meat to soften or putrefy a little in the sun, every people has used it; though it appears not to have been so common in the days of Homer, for he gives to some of his heroes, amongst other appellations indicative of great strength and ferocity, the name of *raw-eater*. Much of the vital elements are, no doubt, lost in the process of cooking, and Dr. Philip, in conformity with this remark, says, " However imposing the plans of concentrating much nutriment in small compass may at first view appear, we may be well assured that, in such concentration, something is taken away from what nature designed for our food, which is useful to us."

The author, in the next place, treats on the digestibility of the vegetables commonly used as food. Fresh vegetables, on account of their tendency to ferment, are, on the whole, he says, injurious in indigestion. Peas, beans, cabbage, and waxy potatoes, he has found the worst: mealy potatoes, turnips, and broccoli, among the best. Fruits appear to be difficult of digestion in proportion to the quantity of mucilage contained in them; though none of these is so much so as " the cold fruits, melons, cucumbers, &c." Most stomachs, it is said, " bear acids better than acescents."

With respect to drink, the author says, " in health, when the various functions are in due proportion, little liquid is required with the food." Thirst often exists when the stomach is surcharged with vitiated fluids. Of the propriety of drinking at meals, he says, that we should not, in conformity with custom, drink when we feel no desire for it. If there be no thirst, he says, we may be assured that the food already possesses the proper degree of moisture, and that any addition to it will only dilute the gastric juice, and consequently enfeeble its solvent power. We know several persons who attribute their recovery from habitual indigestion, to abstinence from drink at meals, even though they felt much desire for it. Dyspeptics are often induced to drink to an enormous amount, by the thirst dependant on the morbid irritation of the alimentary canal, which is attended with a want of the due secretions. The author introduces here some very interesting remarks on the qualities of fluid aliments; from which we shall transcribe the following.

"We have seen, that that part of the food which lies next the stomach having duly undergone the action of the gastric fluid, is moved onwards towards the pylorus; while that next in succession is, in its turn, applied to the surface of the stomach, where it excites a further secretion of gastric fluid, undergoes its action, and in like manner is moved onwards towards the pylorus.

"That the motions necessary for these purposes may be readily performed, a certain degree of moisture is necessary; but, if the contents of the stomach be wholly fluid, it is evidently impossible that such a process can go on with any degree of precision. The fluid cannot be so changed as to present a constant and regular succession of food, comparatively fresh, to the surface of the stomach; there will not, therefore, be the same stimulus to excite to a continued secretion of gastric fluid, and what is secreted will be too easily diffused through the liquid contents of the stomach to make the proper impression on any one part: the same must necessarily happen to the more digested part in its passage to the pylorus; it must be more or less diffused through the other contents of the stomach: in short, no part will be duly digested. The gastric fluid, being too much diluted for its function, will be rather diffused through the contents of the stomach than neutralized by them; hence, the appetite is never perfectly allayed, and little nourishment afforded. Thus the effects of liquid food tend to confirm the view of digestion afforded by the facts which have been laid before the reader."

Stimulating drinks are considered by the author to be really unnecessary; but, he remarks, the use of them in one form or other is so general, and, in most people, the habit which requires their continued use is so fixed, that they can seldom be wholly withdrawn, except in very early life, without doing more harm than good. He enters into a discussion of some length on the qualities of alcohol in its various forms, and dwells particularly on the essential difference in the effects of it, when it has been distilled, and those it produces in the state in which it previously existed in fermented liquors: an observation which has been made by many practitioners, who thence, and from the superior intoxicating quality of a given proportion of alcohol when distilled to that it possesses when in wine, have supposed that the alcohol exists in an elementary form only in merely fermented liquors, and is really formed, as alcohol, by the agency of fire; but this notion seems to be opposed by the fact that alcohol may be distilled from wine at the temperature of 56° (Fah.) in a vacuum, in the same quantity as it might be obtained by fire. Dr. Philip notices the qualities of the different sorts of wines and fermented liquors, without, however, adducing any thing peculiarly interesting. We have the same remark to make on what he says on tea and coffee; except that there are many persons who, like ourselves, would, from gratitude perhaps, say more in their favour than he has done.

The temperature of the drink of dyspeptics should not, according to the author, be above that of the body. The efficacy of a draught of hot water an hour or two after dinner, may be here urged; but, Dr. Philip says, "the relief thus obtained is, like that from distilled spirits, generally compensated by subsequent debility, which, although inconsiderable at first, is often at length severely felt."

With respect to the frequency of meals, the author says, "it has appeared to me that, with the generality of dyspeptics, to take three moderate meals in the twenty-four hours is the best rule. A few find four meals better. The last meal should always be taken a little before bed-time, and should never, particularly after the disease has continued for some time, consist of animal food." Dr. Philip remarks, that there are cases where food may be necessary more frequently than four times in the twenty-four hours. Sometimes the stomach can bear so little at one time that, were the usual intervals observed, due nourishment would not be received: but, he adds, when it is necessary to eat very often, every care should be taken, by recurring, as soon as possible, to a better plan of diet, to prevent the habit of very frequent eating being formed. Among the evils of this practice, an artificial want arises, and, if the patient is not continually taking food, he feels a sense of sinking which persuades him that its constant reception is necessary to his existence.

The author notices, concisely, the effect of different sorts o food on the bowels as well as on the stomach. Much anima food, he remarks, is apt to produce costiveness; but it is bette to take aperient medicines than to disorder digestion by an improper diet. The French practice of employing glysters commonly in these cases, in preference to medicines administered by the stomach, is very advantageous, but it is too repugnant to the English taste to come into very general use with us. Dr. Philip says, "I have in several instances seen advantage from eating household bread mixed with a certain proportion of rice, previously softened by boiling. This admixture, contrary to what might be expected, renders the bread aperient." He concludes this subject by remarking, that, upon the whole, the diet best suited to the stomach is generally found best for the bowels also.

Exercise, and the other points of regimen, are the subjects next discussed. There remained but little to be ascertained on this occasion; we are not, therefore, disappointed in not finding any thing remarkably novel or peculiar to the author: what he does adduce is, however, much to the purpose. We are pleased at finding him say a few words in favour of friction on the surface of the body, in persons too languid to exercise themselves; though there is reason to fear that, if this luxury were to be generally indulged in by the sick, it would soon become vulgar, and counteract what the French are now endeavouring by instituting public gymnasia.

We now arrive at the more expressly medicinal measures for the treatment of indigestion. The author commences his remarks on these points by the following outline of the practice he inculcates.

"It appears from what has been said of the nature of the first stage of indigestion, that it arises from the debility of the muscular fibres and nervous influence of the stomach and bowels. While we are, by a proper regulation of diet and exercise, endeavouring to prevent every cause which may increase their debility, it is necessary, by the aids which medicine affords, to endeavour more directly to restore their vigour.

"The medicinal treatment of this stage may be divided into that proper while the disease is confined to the stomach and bowels, and that which becomes necessary in consequence of its having spread to other parts.

"We have seen that, although the causes of indigestion seem, some to act on the muscular fibres of the stomach, and some on its nerves, yet these powers are so connected in their functions, that whatever debilitates the one, in a greater or less degree, necessarily affects the other. We shall find that a similar observation applies to the means of relief. Whatever tends to restore a healthy nervous power to the stomach, tends to form the food into that substance which is best fitted to excite the muscular fibres of this organ; and whatever excites the natural action of these fibres tends to relieve the nerves from their load, and, in the most favourable way, to bring into contact with their extremities the food on which their powers are to be exerted.

"Although some of the remedies seem to operate more in the one of these ways than in the other; as I wish to avoid nice distinctions, it will be better to include the whole under one head, in an inquiry respecting the proper use of stimulant remedies in this disease; and I shall attempt no other division than the simple one of those which act directly on the stomach and bowels, and those which influence them through other parts.

"But, besides the means which alone deserve the name of curative, others, to be regarded as preparatory, must occasionally be employed. To give the curative plan the best chance of success, it is not only necessary to remove the remote causes and prevent their re-application, but, as far as we can, to remove the more immediate effects of these causes, and thus bring the digestive organs into the circumstances most favourable to the operation of that plan.

"When we are consulted by those labouring under indigestion, we generally find the stomach and bowels loaded. It is necessary, in the first place, to relieve them from some part of this load, and, as far as we can, to correct the properties of that which remains. On this account, we frequently find it advisable to commence the treatment by an emetic, followed by some mild aperient. It should be our endeavour, by an attention to the proper rules of diet, to prevent the necessity of repeating the former of these; and the latter, we shall find, only makes part of the general plan, as far as it is necessary for the regular and free action of the bowels."

The first steps in the course of treatment are to be directed to the restoration of the digestive functions to the natural state, as nearly as the debility of the organs will permit. We may just mention, that the means recommended by the author are aromatics, the alkalies, (sometimes ammonia,) saline draughts in the state of effervescence, camphor, small doses of opium occasionally, to alleviate casual pain; and, in short, a mode of treatment not remarkably different from that of the generality of practitioners.

The means just alluded to are chiefly intended to remove the present symptoms of indigestion; the author, in the next place, considers how far medicine can assist the due regulation of diet and exercise in preventing their recurrence. It will immediately be anticipated that the bitter vegetables are the medicines from which the author expects the most beneficial effects. He thinks the most important distinction to be made of these substances, is into those which have marked stimulative properties, and those which are comparatively devoid of them. The former are to be employed, in general, in the earlier part of the first stage of indigestion; the latter, when there is any inflammatory condition present. The author's opinions of the relative qualities of the bitters differ from those of most other practitioners: he says, "of those in common use, camomile, bitter orange-peel, and wormwood, seem to be most free from any stimulating quality. Calumba possesses this quality in a greater degree than gentian and cascarilla, and the Peruvian bark possesses it in a greater degree than any other." Leaving out the Peruvian bark, we should just give a reverse statement; that is, attribute to substances possessing such a large quantity of pungent essential oil as orange-peel, camomile-flowers, and wormwood, greater stimulant properties than to calumba; a wood containing no essential oil, hardly any resin, nor any pungent flavour. The author says nothing of quassia, which is the bitter we should place the lowest in the scale in regard to immediate stimulative properties. We speak from practical observation, as well as from the chemical and sapid qualities of the medicines. The author mentions in very favourable terms a cold infusion of cinchona; but he is, we believe, very far from being correct when he asserts that this bark has been very generally banished from the treatment of this disease.

Dr. Philip believes that the notion of the long-continued use of bitters being injurious, is not true: it has chiefly arisen, he thinks, from their effects in gouty cases, and that "the bad effects which ensue should rather be ascribed to the prevention of regular gout, than any direct effect of the medicine." This reasoning is not quite satisfactory, even on the principles of the author's own etiology of gout: for, if gout be prevented by the alleviation of its cause, the indigestion, its prevention should not be productive of ill consequences. The bitters, we must suppose, according to the author's principles, prevent gout only by alleviating or removing indigestion. If it be said that the bitters alleviate the indigestion so far as to prevent the occurrence of one of its more severe effects, the gout, and not avert many other of its ill consequences, we have this dilemma presented to us,—whether it is not better to let indigestion, in some cases, become very bad, in order that gout, where there is the predisposition to it, may be produced, and thus the indigestion and the rest of its effects obviated, than that we should attempt to palliate it.

Astringents, the author says, are less generally adapted to cases of indigestion than bitters, on account of their tendency to increase the inactivity of the bowels: "some of them, however," he adds, "are medicines of such value, that we often find it advisable to employ them, although at the expense of correcting this effect." The best of this sort of medicines is, he thinks, iron, especially its carbonate, "provided it can be taken in rather large doses without oppressing the stomach." Next to this is the sulphuric acid, which is particularly serviceable "in those cases where sweating, which is not unusual, is too easily induced by exercise;" and, he remarks, that it appears from his experiments relating to the circumstances which influence the state of the urine, "that the saline matter, secreted by the skin, is not so certainly thrown off, even by profuse sweating, as by a free insensible perspiration." Of the sulphate of zinc, a medicine, we think, of very great value, the author says, "It may be given at later periods than iron, but it requires caution; and, if its good effects do not soon appear, should be laid aside. It is one of those powerful agents which must always be employed with some degree of suspicion."

Sarsaparilla appears to Dr. Philip to "hold a much higher place among the remedies of this disease than is generally supposed, but it is not to its early stages that it is best suited. From its mucilaginous property, it is apt to oppress the stomach. It is in protracted cases, where general languor of the secreting surfaces has supervened, that it is most useful."

The reader will probably be surprised that we have proceeded so far without saying any thing about what has for some years past been the English *panacea.* The author has a decided aversion to the administration of mercury: he says, "I believe its use is always injurious in the period of the disease we are now considering; that is, while the derangement is confined to the alimentary canal."—"I believe we may say," he adds, "without hesitation, that it is never to be used in this disease for the mere purposes of an aperient."

When indigestion has become attended by derangement of the function of the liver, the author acknowledges that the use of mercury is advisable; but he thinks that "it is not to be so given as to be received into the circulating system."—"Mercury," he adds, "when thus introduced, has the property of more or less exciting all the secreting surfaces; but their excitement is supported by it at great expense to the constitution, and, when long continued, seldom fails to impair its powers. No practice can be worse than that which unnecessarily risks this effect. Now, experience has amply proved that the deranged action of the liver can, in consequence of the sympathy which exists between this organ and the alimentary canal, be corrected in the first stage of indigestion by the local effect of the mercury on the latter."

In many cases, one or two doses will produce a secretion of healthy bile, which may be thus supported by the other remedies; and "in the more obstinate cases indeed, where the disordered state of the liver constantly recurs at short intervals, it is better for a certain time to give a moderate dose at stated intervals, by which the alimentary canal will suffer less; as a smaller dose is required for the prevention of this state than for its removal. But, even in these cases, this practice should not be long pursued, without trying from time to time how far the powers of the constitution are sufficient without its aid."

We cannot follow the author, in our analysis, through all his remarks on the use of this medicine; the rest consist of minute observations, of only secondary importance to the general precepts which we have adduced: we should, however, mention, that he concedes to the favourers of this remedy that the blue-pill, administered every second or third night, may be proper when the affection of the liver has supervened very early, and when it is the primary disease, or that which supports or aggravates the indigestion.

Some of the mineral acids are, Dr. Philip thinks, the best substitutes for mercury. "In some cases," he says, "we shall find the dandelion an assistant to it, and under certain circumstances capable of being substituted for it."

The author, after this, enters on the consideration of the treatment proper for the second stage of the disease. We must confine ourselves to a general account of this and the subsequent parts of the work: it is not possible to abridge, in a manner consonant to the limits of our Journal, practical discussions of this kind, without in some measure doing injustice to an author, by omitting to notice many more or less important and very essential observations. We have already, we think, cited what is sufficient to justify the opinions we have expressed respecting the value of the book, and have thought it better, for our own purposes as well as for the interests of the work, to give a tolerably comprehensive account of the most important parts of it, and merely a slight general view of the rest, than to produce only a very imperfect and unconnected notice of the whole.

The chief alterations requisite in the treatment of the "second stage," where there is inflammation of the stomach and generally of some part of the liver, must be sufficiently obvious. The more stimulant kinds of bitters and aromatics are injurious, and the patient often thinks that his disease admits of no relief but from aperients, and particularly mercurial aperients; of the good effects of which he is always sensible, and consequently is very apt to fall into an excessive use of them. These are considered by the author as only part of the means to be employed. The treatment is not to be regulated by an attention to the inflammatory symptoms alone: the more powerful antiphlogistic measures, as a very low diet, general blood-letting, &c. are but rarely proper, "unless the inflammatory symptoms, as sometimes happens, increase to those of active inflammation." "The less stimulating of the tonic means employed in the first stage are still indicated."

Another alteration in the mode of treatment results from the greater attention to the sympathetic affections that becomes necessary in the second stage. Particular care is, also, now requisite to avoid exhausting the patient's strength, which is necessarily more impaired in the second stage, whilst the means of relief are, at the same time, of a more debilitating nature, and the power of restoring the vigour of the body by nourishing diet suspended. An account of the particular means of fulfilling those indications does not accord with our present purposes:

the merit and chief utility of the author's considerations on them consist in the appropriate application of common measures to the various states of disease, not in novel remedies; and they cannot be shown without entering into extensive details. We may, however, just remark, that the author is inclined to resort to the use of leeches, and let the patient bear, for some time, the depression resulting from the deprivation of stimulants to which he had been accustomed, than to attempt to remove the inflammation suddenly, by copious and frequently-repeated blood-letting. In the less inflammatory cases, blisters sometimes relieve the symptoms without the aid of leeches; but this relief is, in general, not permanent, unless the more stimulant part of the treatment is abandoned. He has not found it necessary to have the patient abstain wholly from wine, though the quantity of it should be lessened; and it may be drank diluted, except in a few cases attended with a considerable degree of hardness of the pulse, when a diet wholly vegetable, besides, has been very advantageous. Mercury is here, in general, beneficial. The author's mode of administering it is peculiar to himself: he says,

"I have generally given a grain of the blue-pill, sometimes only half a grain, twice or three times in twenty-four hours, till the secretion of the bile appeared to be healthy, repeating these doses when it was again disordered; and by such doses, which may appear to many little better than trifling, I have seen the bile gradually restored to a healthy state, when larger doses had been employed in vain. They not only often succeed where larger doses fail; but the change, in proportion as it takes place more slowly, seems generally to be more permanent.

"The correction of the state of the bile, however, is but one of the effects of such a plan. Along with its improvement, the skin generally becomes relaxed, and of a proper temperature; the pulse more dilated; the colour and expression of the countenance better; and, in particular, that expression of languor so peculiar to the advanced stages of the disease, abates. As all these changes depend on a common cause, and consequently take place together, the state of the bile, which should from time to time be ascertained, is a good indication of the general effects of the medicine."

He has always discontinued the use of mercury when the slightest affection of the mouth appeared, and any degree of salivation has generally seemed to him "to do more harm than good." When the blue-pill, in the form above prescribed, disorders the bowels, it should be combined with the extract of poppies, hemlock, or hyosciamus, especially the latter, in doses of not exceeding a grain of the extract, two or three times a-day. When the mercury cannot be borne in any way, the mineral acids are to be substituted for it; and the author thinks that the external use of them, as recommended by Dr. Scott, is much more powerful and efficacious than the administration of them internally.

After discussing particularly these and various other remedial measures,—amongst which, the nitrate of potass, taken in a considerable quantity of water, with a proportion of mucilage, is spoken of with much approbation,—the author notices the curious circumstance of the stomach becoming relieved, and performing its functions with tolerable energy, when the sympathetic affections have attained a certain degree of severity, and now engage all the attention of the patient, as well as require the especial consideration of the medical practitioner. Sometimes, on the development of this change, but little is complained of besides great general debility; and it is not uncommon for patients to express their surprise that they should be so weak when the stomach performs its office so much better than when they felt very little of this general debility; and the author adds, what is very remarkable, that he has found the debility most obstinate when least complicated with determination to particular parts, provided change of structure has not taken place in the latter case. The effects of the disease have here very frequently been augmented by the means of cure, when mercury has been employed to any considerable extent; and yet, from mercury, under these conditions, temporarily affording relief, by restoring the suspended secretions or correcting such as are morbid, its aid is still very commonly resorted to. It is more frequent, however, for the disease to manifest itself particularly in one organ than to produce merely this state of general debility. The treatment should, of course, vary essentially according to the presence or absence of chronic inflammatory disease in the parts secondarily affected. When there is no local inflammation, blood-letting is out of the question. The pulse is generally hard; "and this hardness," the author says, "is relieved with difficulty; because, not depending on the affection of any one part, local evacuations influence it but little, nor are they at all the appropriate remedy; and the general state of debility admits of but a very cautious use of those which produce their effect on the whole system."—"The pulse," he adds, "must be softened by a mild diet and medicines which excite the secreting surfaces." Mercury is objectionable, for the reasons already mentioned. "The moderate use of saline medicines is among the best means in such cases. It would surprise any one, whose attention had not been particularly directed to them, to observe the effects which a diet composed wholly of vegetable substances and milk, if the stomach can bear it, often produces in those labouring under this form of the disease, who have been vainly endeavouring to support their strength by a large proportion of animal food. It has long been admitted, indeed, that such a diet is sometimes useful in cases of debility."

The medicine from which the author has seen the most beneficial effects arise, in the generality of cases, is sarsaparilla; "which often seems," he says, "by its continued use, to give a general tendency to greater freedom in the secreting surfaces. I have frequently seen it, by its mild stimulant and tonic powers, succeed where every thing else had failed."

A favourable climate, that is, one where the air is dry, fresh, (but not very sharp,) and clear, is particularly beneficial: the author speaks of Malvern, as a place of residence, with particular approbation. A mere change of air is often very advantageous.

We shall not attempt to follow the author through his particular discussions on the treatment of the secondary affections: his precepts are conformable with general principles of pathology and therapeutics, and more or less similar to those commonly prevalent. We turn over, therefore, forty or fifty pages, to the chapter on the "third stage of indigestion," (that is, the state where "change of structure" has taken place,) which commences with the following general remarks:—"The stomach is less liable to change of structure than most other organs. This change, therefore, although sometimes taking place in it, is much more frequent in the parts with which it sympathizes."

By "change of structure" and "organic disease," the author means such change of structure as is apparent after death. The comparative freedom of the stomach from this may, perhaps, be in a considerable degree attributable to the circumstance that the sympathetic affections so frequently suspend or remove the irritation of this organ.

The diseases arising from neglected indigestion are so various, the author observes, that to give any thing like a satisfactory account of them would require a treatise of greater extent than the whole of that now before us; and, he adds, "a superficial account would be worse than none:" he therefore considers only those cases, to which, from their great frequency in this country, his attention has been particularly directed,—that is to say, "the pulmonary affections produced by a disordered state of the digestive organs." His dissertation on these subjects is but an amplification of those he had already produced in a Paper, published in the seventh volume of the Transactions of the Medico-Chirurgical Society, on Dyspeptic Phthisis; and in one read to the Royal Society in 1816; and some observations in his "Experimental Inquiry," on the efficacy of galvanism for some species of asthma. We need not, therefore, occupy ourselves now with what has been for so long a time before the public, considering that the present additions are principally details intended to illustrate the author's former observations.

---

*Observations on some of the General Principles, and on the particular Nature and Treatment, of the different Species of Inflammation; being, with Additions, the Substance of an Essay to which the Jacksonian Prize, for the Year* 1818, *was adjudged by the Royal College of Surgeons.* By J. H. JAMES, Surgeon to the Devon and Exeter Hospital, and Consulting Surgeon to the Exeter Dispensary. 8vo. pp. 328. T. and G. Underwood, London. 1821.

THIS work is concise in its details, and exhibits an extended view of the phenomena, causes, and connexions, of the subjects which it embraces. The therapeutical precepts are briefly, but very correctly, laid down, and unencumbered by superfluous matter. The mode in which the subject is arranged is novel; and, although we shall feel ourselves bound, in the spirit of independent criticism, to dissent from the principle on which it is founded, yet we consider it, upon the whole, managed with considerable ingenuity. We can, therefore, and chiefly on account of the condensed view which it exhibits, recommend it as a useful manual of the class of disorders which it comprehends. But, at the same time, we can confidently say, that the arrangement here proposed will never obtain any thing like general adoption.

The basis of the classification chosen by the author, and which we believe to be his own, is a characteristic of inflammations much insisted upon by the justly celebrated JOHN HUNTER, whose peculiarities of reasoning he has in a considerable degree imbibed; and, instead of attempting at least the explanation of phenomena, he in many instances has been contented with the use of terms that mean no more than the expression of their existence in the disease which he describes. Upon these grounds, therefore, we consider his pathology to want depth.

It was, perhaps, the greatest fault of the master of British pathology just alluded to, that he substituted expressions which implied a specific and an appropriate intelligence to the parts concerned in the phenomena which he described, and which, by easily solving the difficulties that could not elude his penetration, and from coinciding in some degree with his doctrines respecting the seat of vitality, become eagerly embraced by all the followers of his school; and the more so, because it carried them at once and without difficulty to the *ne plus ultra* of causation. Although no one can be more impressed than we are, (and we speak it with the warmest feelings of admiration,) with the wisdom of the intelligence that regulates the animal system, yet we cannot conceive any thing more absurd than the supposition of a peculiar part of that intelligence to be allotted to the various organs of the body. The Supreme Intelligence operates by more simple and more sublime causes; and what we, in the limited state of our faculties, view as a law of nature, may, nevertheless, be itself the result of a still more remote, and surely not less beautiful, principle giving rise to and regulating that and many other phenomena both in the material and animal creation. To assign to a particular law of nature all the sensible phenomena which our confined views of her operations can but imperfectly explain, would be to multiply those laws to a degree incompatible with the simplicity which, in the spirit of true philosophy, we ought to consider one of the chief characteristics of her beautiful agencies. But we must proceed with the work under consideration.

The author divides his work into *two* parts. The *first* contains observations on the general principles of inflammation: namely, the state of inflamed parts;—on the accordance of the general and local symptoms of inflammation;—on the causes which, affecting the system generally, influence its progress;—on the purposes and uses of the different modes of inflammation; —and on mortification.

The *second* part is occupied by the different species of inflammatory action; considered in detail, according to the classification which he has been led to adopt.

We shall follow the author through the successive chapters of his work, and endeavour to present our readers with a critical analysis of the matter which they contain.

The *first* part is introduced by a view of the principles on which a classification of inflammations may be founded. The necessity of a just arrangement is discussed along with the difficulty of forming one against which formidable objections may not be urged. The author remarks. "There can be no question that in the study of every science, a just arrangement will greatly facilitate the acquisition of knowledge, as well as enable us to recollect what has been learnt, or refer to what we wish, with greater readiness, and will probably lead to more correct and enlarged views respecting its various branches. It is one of the principal objects of this essay to *attempt* such an arrangement in that extensive and important department of medical science, which comprehends the various kinds of inflammation."

He pleads as an excuse for the imperfection of the classification which he has adopted, "the want of permanence in the characters of diseases; from their liability to be affected by a variety of circumstances, from which the subjects of natural history are exempt, from the essential differences in their nature from any of these subjects, as well as from the peculiar difficulties which oppose our observations; the same precision is not to be attained in forming our divisions, which in them is so admirably supported throughout." While we fully acknowledge the difficulty of classifying the various phenomena of inflammation, or those peculiar lesions of textures, which, as cognizable by the senses, have had the term inflammatory affixed to them; and while we are fully aware that no state of disease, notwithstanding the almost tangible shape it assumes, and the manner in which it can be subjected to close observation in its various stages, presents fewer immutable characters on the one hand, or points of essential difference on the other, to guide an arrangement into orders, genera, and species, than the one under consideration. Still, the difficulty which is thus sensibly felt, in our nosological attempts, is not confined to disease alone. Every attempt to classify even the works of nature is beset with the same perplexity; and in every system, whether founded on natural or artificial distinctions, or more or less partaking of either, there will be found both genera and species that cannot with propriety be assigned to any one order more than to another. It has been correctly said by a naturalist of deserved celebrity, that nature did not form distinct genera; and we may say without fear of contradiction, that her operations, as they are manifested in disease, cannot be invariably assigned even to distinct species, so much do the characters of one species of ailment insensibly glide into those of another, according to the states and peculiarities of temperament and habit. Such being, then, the mutable quality of this class of diseases, the imperfections of the classification which Mr. James has adopted (and which we shall presently exhibit) shall be examined with a due reservation of censure. Before stating his own he gives very briefly the modes of arrangement which have been previously adopted.

"1st. We find," he says, "inflammations treated of as acute, sub-acute, or chronic; but these are merely different stages of the same disease in many instances." This is certainly not altogether the arrangement which has been longest in use, at least in this country. Acute and chronic, tonic and atonic, sthenic and asthenic, &c. were usually assigned, and certainly in a manner sufficiently vague, to all inflammatory actions. The imperfections of such an arrangement became apparent as pathological knowledge advanced. It was then easily perceived, that such terms merely indicated states of inflammation, marked by a comparative degree of intensity; and as nature did not invariably exhibit the different species of this class of diseases, either in a state of positive activity nor its opposite, but that they did actually exist, from the maximum of intensity, down through its various degrees, until they insensibly arrived at the lowest form of chronic or ataxic inflammation, or capillary derangement. Hence arose the necessity of employing a word that might convey an intermediate idea, and which might more precisely express in many cases of inflammatory action, the actual degree of the disease present. The term sub-acute became consequently in use among continental pathologists, and ultimately among ourselves.

Mr. James next very justly considers, that, what has been termed the adhesive, suppurative, ulcerative, or gangrenous processes, furnish no fit grounds upon which an arrangement of this tribe of diseases ought to be founded. Nor do the terms phlegmonous, erysipelatous, or gangrenous, afford a better basis.

The former classification indicates stages only, very frequently of the same type of disease; the latter, states of the same disordered action which insensibly glide into each other, and which, when opposite in their characters, are indebted to the tissue in which they are seated, or to the peculiar temperament of the individual, for their points of dissimilarity.

The author next proceeds to examine the classification adopted by Smith, Pinel, and Bichat; namely, that referring them to the elementary tissue in which they occur.

To this system, which, while we acknowledge its imperfections, we consider capable of being made the basis of an arrangement infinitely superior to the one he has given us, the author urges the following objections:

"1st. That different kinds of inflammation are liable to occur in the same tissue. 2d. That the same kind of inflammation is often met with in different tissues. 3d. That the same inflammation shall be transferred from one to another; a position, I must grant, less susceptible of direct proof than the two former; but either are sufficient for my purpose."

In our opinion, these objections are not valid, and we deny the positions which the author takes in support of them. We shall examine them a little more closely. He continues,

"1st. That different kinds of inflammation are liable to occur in the same tissue.

"*In the skin* there are erysipelas, the various exanthemata, and other cutaneous diseases, particularly ecthyma, which approaches to boil. We have those tumors still more analogous called pinswells, and various other forms, as from blisters, scalds, poisonous substances, chilblains, mechanical injury, &c. which are not erysipelas, and which are not identified, because we choose to call them erysipelatous.

While we consider that the various inflammations seated in the skin assume most diversified characters, and although this texture furnishes him with the best arguments in support of his objection, yet their multiplied forms will more easily be accounted for by the pathologist who looks narrowly into the origin and progress of the phenomena constituting cutaneous disease, and who is not disposed to view things in the mass, nor to judge of them from the character which the superficies may possess, without taking into consideration their more intimate connexions with the adjoining textures. The author knows well that the structure of the skin is not elementary, that it consists of various strata; and that it is the most complicated of all the extended tissues in the body.

If, therefore, inflammation commences in any one of these strata, either in the cutis vera, in the rete mucosum, &c. or if it extends to one or more of them; is it not, therefore, reasonable to expect a class of diseases diversified according to the nature and extent of connexion which the different parts of this texture may assume respecting each other, during the continuance of such disordered action. Thus, inflammation may be originally seated in the lymphatic capillaries, or those which do not receive the red particles of the blood, and which run to, and terminate at, the cuticle. In consequence of this disordered action, they admit the coloured fluid, and become easily perceptible; owing to the state of temperament, and habit of the body, &c. the disease spreads over a greater extent of surface; if its duration be long, or if it heighten in intensity, the subjacent strata become affected, and the cutis vera and the cellular texture may successively partake of the disease. But specific inflammations, such as some of the exanthemata, affecting this tissue through the greater part of its extent, become more equally diffused, according as the inflammatory action is seated in the capillaries belonging to the more superficial layers of the skin; whereas, in those, as in small-pox, for instance, that are seated in the cutis vera, when the habit of body and constitution are tolerably sound, and the inflammation remains confined to that part alone, there is a disposition to form distinct pustules, owing to the nature of its texture, which limits the extension of the diseased action to the point in which it commenced; and it is not until it has reached the adjoining stratum from the influence of peculiar causes, that the character of the inflammatory action becomes changed, when it assumes the appearance usual to such a texture, complicated with the primary morbid affection. But our limits prevent us at present from pursuing the subject through its various pathological relations.

The author further maintains,

"2d. *In cellular membrane.* Besides true phlegmon, we meet with boil, carbuncle, erysipelas, scrofulous abscesses, &c.; affections differing widely from each other."

We would ask the author, whether erysipelas and scrofula are ever primarily seated in cellular tissue, or are those diseases, when met with in such a situation, the result of the extension of the disease from an adjoining but different texture? And whether the majority and indeed all inflammations, when seated in the same tissues owe not their points of dissimilarity to peculiarity of constitution and habit, and to the circumstances which give rise to, and influence them, in their progress?

And even granting what is not very evident, that scrofulous abscesses may sometimes arise in cellular substance, does not the word scrofula imply a peculiarity of constitution that accounts for such an anomaly? But all chronic abscesses are not scrofulous, at least, at their commencement; the latter term has been too vaguely employed, owing, perhaps, to the circumstance of the greater tendency of abscesses to become indolent and to assume the modification of diseased action peculiar to the constitution, when they take place in individuals of such a diathesis. We are inclined to believe that the greater part of those disorders to which the name scrofulous has been applied, are not actually of this character; while we are of opinion that those which possess originally the strumous features, commence in the glandular texture, where, from the nature of its conformation and connexions, the adjoining cellular tissue more readily partakes of the disease. It is generally admitted, that in constitutions thoroughly scrofulous, inflammations occurring in any of the textures, and commencing in the usual manner, and from the wonted causes, will put on the constitutional peculiarity. But surely such disorders cannot, strictly speaking, be considered as scrofulous, since they assumed that appearance from the constitutional influence during the progress of the disease, and not from any original peculiarity. The author goes on to observe that,

"3d. *Mucous membranes.* It is admitted ulcerate in one point, effuse lymph in another, and secrete pus in a third; they are the subjects of catarrh, dysentery, angina, croup, of various forms of inflammation of the bronchiæ and of the intestinal canal, described by Badham, Hastings, Abercrombie, and others."

It is well known, that the character, and even the constitution of mucous membranes, vary considerably in the different organs to which they extend; they are supplied with nerves frcm different orders of this system, according to their situation, and they therefore possess varying degrees of sensibility in proportion to the predominance of either. Indeed, their peculiar conformation is in no small share owing to the intimate manner in which the extreme nervous febrillæ are interwoven with the capillaries. Are we not, therefore, prepared to expect disorder in these extreme vessels proportionate, both in degree and kind, to the impressions made upon the nervous system, either of the part or upon the body generally?

Are we not to expect that a diseased state of action in these capillaries will be accompanied with phenomena, differing in very variable proportions, corresponding to the ratio in which the ganglian or the other order of nerves may be distributed upon their surface? And is not inflammatory action, when seated in them, to be expected to assume different degrees of intensity, as it does in other textures, according to the causes, and the modifying influences operating upon the disease, whether arising from fortuitous circumstances, or from those that are peculiar to the individual?

Respecting the assertion contained in the last quoted paragraph, that mucous membranes ulcerate, a more intimate view of the subject will, we are convinced, satisfy Mr. James that such is actually not the case. The ulceration which takes place in the surfaces covered by this class of membranes, arises in consequence of the inflammation extending from contiguity to the immediately subjacent cellular texture, which in the progress to the ulcerated state detaches the mucous membrane. Of this there are many instances continually occurring in intestinal diseases, seated chiefly in the mucous coat, in which this membrane is often detached in the form of flocculi and ulceration extended to the external covering. This was too important a fact in pathology to escape the penetrating view of John Hunter, who has expressly said that mucous membranes never ulcerate, nor is such a process observed in situations covered by this tissue, until the inflammatory action has extended to the cellular texture connecting the mucous to the subjacent tissues. It is unnecessary for us to occupy more of our time in the examination of the objections urged by this writer against what, in our opinion, may be made the basis of an arrangement affording more distinct and permanent marks to guide the nosologist than any other that can be chosen in the present state of our knowledge. We certainly allow that constitutional causes may exist which in a great measure determine the nature of the inflammatory action, in whatever texture this disorder may be seated, or from whatever cause it may originate: yet we contend, that such an influence modifies only, without giving rise to a generic difference in the disease when it takes place in the same texture. Erysipelatous inflammation may affect the membranes of the brain: rheumatism may invade the peritoneum; and several of the exanthemata, instead of appearing on the surface of the body may attack the mucous coats lining the intestinal canal on the bronchiæ and air cells; but the character of the disorder, however it may be modified by the specific constitutional ailment, will be most materially changed by the texture in which it is situated.

On the local symptoms of inflammation, the author is extremely brief. After explaining the essential characteristic—the redness of the part—in the usual manner, he goes on to observe upon the temperature of the inflamed texture.

"The *heat* may, by rational analogy, be explained, on the supposition that the actions of the part are increased, whether of its nervous or vascular system, or both; for it probably proceeds from an increased incitement of the former, supported by an increased circulation in the latter.

"The sensation of heat is to be distinguished from its formation; it seems to be increased, whether the actual heat be so or not, and in no proportion to that, and is very remarkable in gangrene, in which the nerves are undergoing destruction. In some degree, it may be owing to the great increase of the sensibility from which any given cause will produce an increased effect, and hence an ordinary temperature will give *pain.* It has been maintained that parts which naturally do not perceive heat, do not when inflamed; that the mode of pain in disease is analogous to the mode of sensation in health. But this is not to be admitted without reserve; for the pleura gives the sensation when inflamed, and in enteritis it is excessive."

This, upon the whole, is as satisfactory a passage as any we find connected with the exposition of the local phenomena. His observations upon the pain, or uneasy sensations, which form one of the symptoms of inflammations, are as follows:

"The *pain,* it may be presumed, is a consequence of an unnatural impression being made on the nerves; but the mode of pain will vary according to this impression, which depends upon the nature of the cause, the part, the kind, and stage of inflammation.

"It has been explained by the sudden tension of the nerves, from the swelling, or the pressure made upon them in unyielding parts; but then it should be in proportion to this tension or pressure, which it is not. The pain in the inflamed pulp of a carious tooth which is not pressed upon, is as great, or greater, than in the periosteum which is. The pain in the free mucous membranes of the intestines is often as great as in the pleura. There is often little pain in large soft nodes, thrown up in a day or two, and a great deal in small hard ones, which are long in forming. Besides, this will not explain the peculiarity of pain; why it should be sharp in the pleura, burning in the skin, and aching in fibrous membranes."

The author, after making these objections, (which, were there not much more valid ones, that could be adduced, might be easily removed by the favourers of the opinions here controverted,) passes on without attempting any explanation of what, we must confess, is by no means capable of an easy solution.

'It may, however, be remarked, that pain, or uneasy sensation of some kind, as often precedes any cognizable disordered action in the capillaries, as it follows as a consequence; and we believe that the uneasy sensation, and even pain, may be most frequently remarked, before any other lesion has taken place in a sensible degree. May not the peculiar sensations constituting the various kinds of pain, be, as it were, the sensible expression of changes induced in the extreme nervous fibrillæ, which produce farther effects upon the capillaries to which they are distributed: and may not the vascular derangement thus induced re-act in its turn upon the nervous cords and their expansions, and by these means prolong or heighten the diseased sensation and action, according to the peculiar causes and influencing circumstances of the case?

Mr. James, in his next chapter enters upon the consideration of what, in our opinion, would constitute the basis of this department of pathological knowledge, were the subject satisfactorily explained; namely, the supposed final causes of inflammation.

A proper elucidation of this much controverted point, by carefully conducted experiments, and by close observation, would go far to settle the various and even vague notions which have been entertained upon this subject. But we are of opinion, that before the explanation of phenomena which constitute disease can be legitimately entered upon, the functions of the parts, during health, in which such appearances are seated, ought to be carefully ascertained. We consider, therefore, that an examination into the functions of the heart in carrying on the circulation, the part it performs in propelling the fluid, and in sucking or drawing it from the large veins; how far the circulation in the capillaries is owing to their own action, and how much is due to that of the heart; and an inquiry as to what extent the office of propulsion, and of suction, performed by this organ, influences the capillary circulation, ought to be instituted. Into this grand field of inquiry our author does not pretend to enter, but passes it by with feelings bordering on despair. After making some introductory observations, he goes on to state, with reference to the opinions entertained by Drs. Phillip, Hastings, and many others, upon this point:

"Under these circumstances, I should feel inclined to sit down contented, without further investigation, if it were not manifest that the doctrines, now maintained with great ingenuity, and founded upon much research, seem to lead, in their *practical application,* to inferences which are at total variance with what I conceive to be the right treatment of the majority of inflammations. For, if it is granted that it depends upon debility of vessels, by consequence it would follow that it should be our endeavour to counteract this debility.

I do not pretend to be able to disprove those facts that have been stated, nor am I entitled to doubt them; but I may be allowed to hesitate before I admit their full application to the phenomena of inflammation in the human subject, and that because they appear to be opposed by a number of *other facts*, not less certain or important; so that, in the present stage of the inquiry, I should feel it the wisest plan to suspend any positive judgment, confessing my own belief to incline to that view of the subject, which considers it to depend upon increased action."

Mr. James afterwards enters upon an examination of the notions opposed to those which he is himself inclined to embrace. We shall only give the objections he urges against the theory of Vacea, which has created so much interest in the present day; but, in justice to our Author's arguments, we shall first transcribe his observations upon the circulation of the blood in the arterial and capillary vessels.

"The heart, it may be inferred from many facts, is not essential to the circulation, at least for a time it may be carried on without, though imperfectly; and it may be presumed that it does not *alone* support it in the human animal, under ordinary circumstances.

Since, then, some other power has been found to do this, the question is, where does that reside? It is maintained that the arteries contract to propel the blood: but if so, either they must contract together, or at different times; if together, they must either do so at the *same* time as the heart, or *alternately* with it; if at the same time, this would prevent the blood from flowing into them at all; if alternately, that would maintain the pulsatory action in the veins, whereas this is of rare occurrence. There is another mode in which they may be supposed to contract, namely, in succession, like an intestine; but, as far as I have ever been able to find, both the dilatation by the impulse of the heart, and the contraction by the *consequent* exertion of the elasticity of the artery, are in immediate succession, and occur at the same moment, at any distance from the centre, which is at variance with this supposition; and, as the elasticity of the arteries diminishes, so as to be almost imperceptible in the smaller branches, in them the pulsatory motion is gradually lost; whereas, if the contractile power operated, it would be continued, nay increased, as *their* contractile powers are stronger than in the larger vessels. Hence, as well as for many other reasons too long to specify here, I should be led to believe that the circulation is not maintained in any degree by the muscular contraction of the arteries; and, I also believe, that their power of contraction is given them for another purpose, quite sufficient in importance, namely, to regulate the quantity of blood conveyed through them, to any part or to the whole.

"It is unnecessary for me here to detail the reasons which induce the belief that a power of propulsion resides in the capillaries; but, as the opinions which have been formed respecting the state of the vessels in inflammation, have originated in some considerable degree from the notions which have been entertained respecting this power in the *arteries* also, it was necessary I should express my dissent in this particular."

Respecting the theory, that ascribes the phenomena of inflammation to debility of the capillary vessels belonging to the part, the Author makes the following observations,—

"We have next to consider the theory of debility and diminished circulation, as maintained by Vacea, Lubbock, Allan, Philip, and Hastings. The opinions of the three former rest chiefly on the hypothesis that the arteries propel blood; and before it can be admitted, it is necessary that this power of propulsion in the arteries should be proved; it is not sufficient that an artery will contract—that it will contract under the application of a stimulus; it should be *proved that, under ordinary circumstances, it does propel the blood.*"

After noticing some of the opinions of Drs. Philip and Hastings, connected with the appearance of a slower motion in the particles of the blood moving through the dilated vessels, owing to their exhausted excitability, Mr. James proceeds to add,

"There is reason to believe that Dr. Hastings may have been deceived in the appearance of the blood remaining stagnant for days, since it retained its arterial colour, or, if not deceived, that this affords a proof of the wide difference between the processes which take place in the lower orders of animals, and the human being, in whom this phenomenon does not occur, as all experience shews; for, if by pressure on the returning vessels of a part inflamed, or not inflamed, the blood be rendered stagnant, it very speedily assumes the venous appearance.

I must also observe, that on the supposition that the blood does move more slowly, it by no means follows that this may not be compensated by increased quantity, so that actually more blood flows through the part. I do not contend for there being an increased velocity of the motion of this blood; I do not contend for there being increased contraction of vessels; but, on the contrary, it is granted that their *dilatation* is a necessary part of the process; but I do not contend that *this dilatation is not essentially connected with debility:* it might as well be argued, that the uterus dilates from debility to contain the fœtus, or the rectum to allow passage to the fæces. No doubt in both these cases the cavities permit themselves to be distended; but having by a temporary suspension of their action, or from the impression of a predominant force, suffered an alteration of their calibre, they immediately resume their usual degree of action on their contents, or probably act on them with more power than before. This may be equally the case with vessels enlarged from inflammation, or in another less exceptionable instance, *i. e.* when the branches of a tied trunk enlarge.

So obviously incapable is the doctrine of debility of explaining the opposite states of inflammation, *i. e.* common or acute, and passive or weak, that its supporters have recourse to the *supposition* of the larger arteries possessing in the former an increased degree of action, but not in the latter, but that the capillaries are in a state of debility in both. Now, of course, if the propelling powers of the arteries are not proved, this must be doubted; and, if disproved, fall to the ground."

The author, after having premised these arguments against the doctrine of capillary debility being the cause of inflammation, proceeds to state the facts and the appearances which tend, in his opinion, to support the opposite theory. He considers, that " the phenomena of inflammation are generally similar to those which occur in processes in which there is every reason to believe that the actions of the part are increased," as in the growth or development of any new part, as of stags' horns, in pregnancy, in infancy, in which cases the vascularity is more evident, and the heat developed in greater quantity. He views an organ inflamed, as one in which there is an excess of life, and as such excess, with the presence of red globules, seem to be requisite to the formation and growth of parts, so he supposes them required to their reparation in disease. We shall allow the author to speak for himself.

"We find the red globules in the cartilage of ossification when the bone is about to be formed, and the vascular appearance which the bone then presents, is precisely similar to inflammation; the process performed is actually the same as that which occurs when the bone does inflame in fractures;—does the natural process depend upon debility ?—is there reason to suppose that the repairing process does so? The actions of the heart are generally increased in inflammation, and the arterial system is commonly more completely filled by its pulsations; the veins returning from an inflamed part are also fuller; if blood be let from either it will flow with more force;—does this look like a diminished or tardy circulation in it? Whoever has felt the throbbings of a paronychia will have a difficulty in believing that there is not an increase of the force of the circulation; but then it is maintained, that this is in the arteries only, and that the feeble capillaries cease to urge it on. Divide the inflamed part, and it will immediately be seen, that the blood pours forth from the whole surface, and in great quantity, more than could have been previously contained in it; but this may be ascribed to the vessels being weak, and unable by their contractions to resist the flow. But observe what occurs; after a short time the open mouths of even considerable arteries cease to bleed a drop, and at the same time the capillaries also contract, and there is no more hæmorrhage: but irritate, stimulate the wound, apply pressure to close the vessels and prevent their bleeding, and there will be no end to the attempts of nature to overcome it, or act in opposition to them."

After noticing inflammations in which the heat is less, and the colour of the blood partakes more of the venous character, which he explains in the usual manner, with those who espouse the same doctrines as himself, and after urging these phenomena as objections to the opposite theory; the Author continues his arguments, and adduces the effects which result from the employment of remedies in support of his opinions; he farther adds, in continuation of this subject,—

"If an inflamed part be pressed upon by the finger, so that the blood shall be forced from the vessels, on that pressure being removed, it will return with great velocity; whereas, if a part be reddened from cold, the contrary will be the case. In the latter we may suppose the circulation to be languid, in the former the reverse."

"The increase of secretions, which generally takes place at some period in inflammation, is another indirect proof."

Respecting the state of secretion during inflammation, as it affects the various tissues of the body, it is a subject deserving closer attention than, in our opinion, has been yet paid to it. We consider that, whoever would distinctly shew the state of the secretions in the various stages and kinds of inflammation, as it may affect the serous, mucous, glandular, and other textures of the body; and how far those secretions are changed in quality and quantity, or to what extent they may be suspended in the different periods, and under the varying circumstances of the same malady, will supply a desideratum in pathological research, especially if performed in a manner which we would expect in the present state of medical science. The opinions hitherto connected with this subject, have been very various, and seldom have been assigned with any degree of precision to the different stages of inflammation, as it occurs in the various tissues. This however is a subject so intimately connected with the state of the capillaries, during the continuance of the inflammatory disorder, that a correct explanation of the one would assist considerably in accounting for the other. Nor is it a subject of so little importance, as the neglect with which it has been treated may lead many to suppose; since it is a material characteristic of several diseases, and in many others ought to assist us in forming opinions of the existing derangement, in giving a prognosis, or in choosing and directing our remedial agents. But we must resume the arguments of the author before us.

"The hypothesis of debility of vessels totally fails to explain one very remarkable phenomenon, namely, that if we divide the enlarged vessels going to an inflamed part, the cause of irritation still continuing, others immediately enlarge, and these even remote from the point where the division is made, as we observe with most facility in ulcer of the cornea: besides, we find the old ones uniting again, or new developed, with a rapidity quite inconsistent with this doctrine.

"Without thinking it necessary to inquire farther into the final cause of inflammation, I should be much disposed to believe that there is an increased action of some kind in the part, in all cases of acute inflammation, even when its powers are weak; and, in so supposing, there is no absurdity, for convulsion is undeniably an instance of an increased action, existing as well when there is much debility as when there is much strength; and that there is increased action we may judge from the results which we see in new growths, in increased secretions, &c.; also that it exists in the minute nutrient and secreting vessels, affecting different modes under various circumstances, which are the nature of the cause, the part, and the constitution; and it is not necessary to define in what it consists. We know that under the influence of a hot sun, a vegetable will grow with rapidity; and we shall not use an objectionable phrase, when we say, that there is an *increased action* in it: yet it is not necessary to connect this with the muscular action of vessels on their contents. We also know, that when an increase of natural secretion is to take place, as from the salivary or mammary glands, the vessels leading to the part dilate: probably it would be wrong to apply either the term increased action or relaxation to them. It may be accounted for by the influence of sympathy felt between the parts where the actions are performed, and the vascular centre."

The author proceeds in his next chapter to treat of sympathy, as it is connected with the subjects embraced in his undertaking. This subject he divides into:—"1st. *Sympathy of immediate Connexion*, which will include the continuous and contiguous. 2d. *General Sympathy*, of the whole with parts, and of parts with the whole. 3d. *Sympathy of Function*." The following passage possesses the fault of the Hunterian school, when attempting to explain many of the phenomena occurring in disease.

"The most simple instance which presents itself of the connexion of inflammation with sympathy, is to be found in cases of injury.

"When a part is injured from any cause, there is an attempt made to repair it by the effusion of organizable lymph, and the conversion of this into animal structure: to effect this, an increased flow of blood into the part, seems to be required, which is accompanied with an increase of its sensibility and temperature, and it may be conjectured that an extraordinary degree of vital energy is manifested in that part. It is from the influence of sympathy that the vessels leading to it consent to enlarge, to transmit the requisite quantity of blood; that the heart itself, if the injury is considerable or important, increases its efforts to send it, and that in some other parts of the body the vessels and the nerves act with diminished powers, in order that a greater supply of nutritive fluid, and the chief exertion of vital energy may be concentrated on that where the new process is going on. The phenomena which occur locally are termed inflammation, those which take place generally constitute sympathetic fever."

The author proceeds to remark the consequences "of the actions excited by sympathy when they exceed due bounds;" here he speaks of "the vessels being gifted with the power of effecting indirectly," by the effusion of pus, and the formation of granulations, that which, owing to the excess of sympathetic action on the part of the constitution, they were prevented from performing in their first efforts. "If, however, the repair has been obstructed by such over-action as now stated, that pus will be of an unhealthy nature; and, if this end cannot be obtained, the last resource of nature, to terminate the struggle between the part and the constitution, is to remove it either by ulceration or by mortification." This is certainly all very true so far as the recognition of phenomena is concerned, but it is no explanation; we believe, however, (unless the spirit has descended upon them of late,) that the desire of further inquiry was never felt by the majority of our profession, believing as they did, and even, perhaps, now do, that a knowledge of what are commonly called pathological facts, was quite sufficient for the purposes of practice. But we apprehend that something more is requisite if we wish to deal with disease in a manner which ought to be sufficiently palpable to the dullest casuist.

The author continues to shew, that the degree and kind of actions sympathetically excited, are much influenced by the nature of the injury, state of the constitution, and nature and state of the part. He afterwards passes on to the consideration of the consequences which result from continuity of surface and of texture, and next, to those arising from contiguity of situation. Here Mr. James takes an opportunity of noticing the opinions of Bordeu and Bichat respecting the distinct and modified vitality possessed by the various organs, and adopts that of Bordeu. He concludes this chapter as follows.

"It is unnecessary for me to dwell either upon the nature of *sympathy of function, or remote sympathy*; but before dismissing the subject of sympathy, it is necessary to state my belief that the phenomena of disease chiefly depend upon the following fact:—That there are some organs which cannot be affected in any way, without all the rest participating in a great degree, and it is intended that they should, under all circumstances, regulate the state of the system; these are, the stomach, alimentary system generally, the brain and the heart; and in proportion as they are capable of influencing others, are they liable to be impressed in their turn. Others again, both impart and receive a less degree of influence; but the rule seems to be, that this is determined by the extent to which their functions contribute to the support of the whole, and it is this which may be denominated general sympathy."

[The continuation of this article is necessarily interrupted by the other arrangements of the Journal, it will be given in the next number.—Edit.]

*A Dictionary of Chemistry, on the Basis of Mr. Nicholson's; in which the Principles of the Science are investigated anew, and its Applications to the Phenomena of Nature, Medicine, Mineralogy, Agriculture, and Manufactures, detailed. With an Introductory Dissertation, containing Instructions for converting the alphabetical Arrangement into a systematic Order of Study.* By ANDREW URE, M.D.; Professor of the Andersonian Institution; Member of the Geological Society, &c. 8vo. price 1l. 1s. T. and G. Underwood, London. 1821.

WE have failed to notice the late improvements in chemistry, in this Journal, except as far as they appertain expressly to medicine and pharmacy, because we could not detail them in such an extent of space as the objects of a Journal especially devoted to medicine would permit us to appropriate to this science; but it must not, therefore, be supposed that we think the knowledge of chemistry of but trivial import to medical practitioners: our opinions are directly the reverse, and if we may appear, on first consideration, to have neglected it too much, we shall, perhaps, be excused, when it is remembered that the whole of the phenomena it embraces are too intimately allied for any merely partial information to be of much avail. A man must be a thorough chemist, and sedulously keep pace with the constant progression of the science, if he pretends to knowledge of it, any further, at least, than relates to some particular object, as (for example) that of medicine. If he is not perfectly on a level with the existing knowledge, nomenclature, and opinions, the writings of the day will be, for the most part, unintelligible to him. LAVOISIER himself, if he could assume his personal identity amongst us, would be almost as much at a loss to understand many of the chemical memoirs of the present period as he would were they written in a style similar to that of the Rosicrusian alchemists.

We notice the Dictionary at present before us, for the purpose of introducing it to our readers as a work which is, we think, very well qualified to supply those who are deficient in chemical knowledge with the information they may desire to attain of the present state of this science. There are many obvious objections to the alphabetical arrangement of a Dictionary as a medium of instruction in a science, and the whole of these are not entirely obviated by the directions alluded to in the title-page to this work: but, to counterbalance these objections, there are the advantages it presents of a ready means of reference, especially useful to persons not very well informed in either principles or details. This volume, being closely printed in a very small type, comprises every thing that is to be expected in such a work. A considerable proportion of it is re-written by Dr. Ure,—but few articles have not experienced some alteration,—and whatever is the production of his pen is characterized by that perspicuous conciseness of manner for which his writings are remarkable. He evidently has a great facility for abstractive analysis, and this has enabled him to present a very great extent of information—much beyond what might have been expected,—in the space of this Dictionary. This production, altogether, presents a very favourable augury of what the author might accomplish in a Treatise on Chemistry, were he to undertake such a work without being fettered and confined by a faulty and imperfect basis, and the plan of such an arrangement as a Dictionary.

Dr. Ure has departed somewhat from the ordinary mode of constructing a Dictionary, for the purpose of a critical examination of many recent opinions and observations; but such conduct is certainly favourable to the utility of the work. It would not have been right to have passed them over unnoticed, and still more improper to have narrated them without comments. We are, however, disappointed in not finding in it any notice of Mr. Bompass's Treatise on Light, &c. Several of the views and inferences in which seem to be supported by some recent observations: the work is, at least, marked by so much ingenuity that we think it should have been noticed for the purpose of discussing its merits. There are many articles in this Dictionary which will render it especially useful to medical men, and which present connected dissertations that could not be introduced in so distinct a manner in a systematic work; amongst which, in particular, are those relating to meteorology, the materia medica, pharmacy, and several matters appertaining to domestic economy, as well as many other analogous subjects, which, though not essential parts of the scholastic education of medical men, are things which should be familiar to them. One of the chief objects of the author appears to have been to show the most important results of the application of chemistry to the arts; and the manner in which he has accomplished this would, alone, give a peculiar degree of interest to his work. When we contemplate these results, we cease to wonder that chemists are such enthusiasts in their pursuits, and, for the most part, so exclusively attached to them. We see in the steam-engine, as it were, a vivification of geometry and mechanics. It spins and weaves, and more equally than any workman, for it suffers neither fatigue nor distraction. In three strokes it makes a shoe: a first cylinder cuts out the materials, a second makes the holes, in which a third drives the pegs, and the shoe is made. It makes sheets of paper of any given dimensions: it would produce them several miles in length, if such were desired. Waggons are carried along iron rail-ways, loaded, returned, and emptied, without any directing power being apparent to the astonished beholder. A ship has been seen to traverse the sea, without sails, oars, and even without sailors. One man to preserve the fire, and another to direct the helm, was its whole equipment. It was propelled by an interior force, like an animated being, like a sea-bird floating on the waves, in the expression of her captain. Sugar may now be made in France in such quantities as to render the importation of it unnecessary: it may be annually prepared to the value of above two millions sterling, by which forty thousand persons may be employed, and a hundred thousand bullocks fatted by the refuse, without an atom of reduction in the former products of the soil. Vinegar is made from wood, spermaceti with horse-flesh, soap with that of fish; ammonia from shreds of cloth; an artificial sort of horn is made from old bones, that may be extended and moulded to any form, and made as thin as paper and as transparent as glass; by a little sulphuric acid, the most impure oil is rendered inodorous and as clear as water; by current lamps we obtain artificial light at a tenth of former cost; the gas-lights are qualified to give a new and highly-improved appearance to our cities and towns; and, were it not for old customs and long-established prejudices, we might obtain many other comforts at comparatively very trivial expence, by profiting from the discoveries of RUMFORD.

